# A tailored lifestyle intervention to reduce the cardiovascular disease risk of individuals with Familial Hypercholesterolemia (FH): design of the PRO-FIT randomised controlled trial

**DOI:** 10.1186/1471-2458-10-69

**Published:** 2010-02-15

**Authors:** Karen Broekhuizen, Mireille NM van Poppel, Lando LJ Koppes, Johannes Brug, Willem van Mechelen

**Affiliations:** 1 Department of Public and Occupational Health, EMGO+ Institute for Health and Care Research, VU University Medical Centre, Amsterdam, the Netherlands; 2 Body@Work, Research Centre Physical Activity, Work and Health, TNO-VUmc, Amsterdam, the Netherlands; 3 TNO Quality of Life, Division Work and Employment, Hoofddorp, the Netherlands; 4 EMGO+ Institute for Health and Care Research, VU Medical Centre, Amsterdam, the Netherlands

## Abstract

**Background:**

Because of a high cardiovascular disease (CVD) risk in people with Familial Hypercholesterolemia (FH), early prevention of cardiovascular disease is important for health gain and cost reduction. This project focuses on the development and evaluation of an innovative intervention aiming to reduce CVD risk by promoting a healthy lifestyle among people with FH.

**Methods:**

This project is designed as a randomised controlled trial in which individuals with FH will be assigned randomly to a control or intervention group. In the intervention group (n = 200), participants will receive a personalized intervention which is a combination of web-based tailored lifestyle advice and personal counselling by a lifestyle coach. The control group (n = 200) will receive care as usual. Primary outcomes are biological indicators of CVD risk: systolic blood pressure, glucose, BMI, waist circumference and lipids (triglycerides, total, LDL and HDL cholesterol). Secondary outcomes are: healthy lifestyle behaviour (with regard to smoking, physical activity, dietary pattern and compliance to statin therapy) and psychological correlates and determinants of healthy lifestyle behaviour (knowledge, attitude, risk perception, social influence, self-efficacy, cues to action, intention and autonomy). Measurement will take place at baseline, and at 3 and 12 months after randomisation. Additionally, a throughout process-evaluation will be conducted to assess and monitor intervention implementation during the trial.

**Discussion:**

Results of the PRO-FIT project will provide information about the effects and implementation of a healthy lifestyle intervention for individuals with FH. Our experiences with this intervention will be indicative about the suitability, feasibility and benefits of this approach for future interventions in other high-risk groups, such as Familial Combined Hypercholesterolemia (FCH) and diabetes.

**Trial registration number:**

NTR1899

## Background

Familial hypercholesterolemia (FH) is an autosomal dominant disorder of the lipoprotein metabolism. Due to a defect of the low density lipoprotein (LDL) receptor gene, plasma concentrations of LDL cholesterol (LDL-C) are elevated[1]. In the Netherlands, approximately one in 300 people is affected with the heterozygous type of FH[2]. In 2003, the Ministry of Health, Welfare, and Sports introduced a national cascade screening program to detect people with FH. The screening program is run by the Foundation for Tracing Hereditary Hypercholesterolemia (StOEH) and through this program, some tens of thousands of people in the Netherlands have already been and are made aware that they have FH[3].

Elevated serum LDL-C and therefore also FH is associated with an elevated risk of premature cardiovascular disease (CVD)[4], which is the disease with the highest burden in disability adjusted life years (DALYs) in the Netherlands [5]. If elevated LDL-C is not diagnosed and treated, the cumulative risk of developing coronary artery disease (CAD) by the age of 60 years is over 60% for men, and over 30% for women[6]. This increased risk does not appear to make people with FH more worrisome[7]. They seem to underestimate their CVD risk [8] and perceive it similar to those in whom no mutation was found[7].

A substantial number of LDL-C mutation carriers are identified through the national screening program. However, a large variety in phenotypic expressions among FH carriers has been found[9]. Environmental factors, lifestyle factors in particular, appear to play an important role in modulating the course of this disorder[10,11]. Until now, research has mainly been focussed on the effectiveness of pharmaceutical therapy, whereas achieving improvement by lifestyle change has hardly been investigated. Large primary and secondary prevention trials with statins have clearly demonstrated the benefit of reducing LDL-C in subjects with high LDL-C[12,13]. Statin therapy is the cornerstone of dyslipidemic management for people with FH, but significant CVD risk persists despite effective LDL-C lowering statin treatment[14]. Two main strategies are of importance to further reduce CVD risk among FH patients: 1) Improvement of adherence to statin therapy, and 2) Improvement of CVD-risk-related lifestyle. Large proportions of individuals with FH receive lipid-lowering statin therapy and still do not achieve LDL-C target levels as stated by the guidelines of the National Cholesterol Education Program (NCEP)[15]. Even though compliance to medication seems high, still 12% of the people with FH never started, and 6.4% discontinued their medication after identification of FH[16]. Additional activities to promote treatment (adherence) have the potential to be effective in reducing CVD risk in these groups.

A healthy lifestyle is an aspect of the treatment of FH with many benefits beyond LDL-C-lowering drugs[17]. Results of primary prevention trials in high-risk persons and secondary prevention trials in CVD patients both show that substantial reductions in the CVD risk can be obtained through lifestyle changes[18]. For example, the INTERHEART study showed that eating fruit and vegetables daily, being physically active regularly and avoiding smoking were effective in reducing the risk of a myocardial infarction by 80%[19].

Altogether, these findings indicate that more comprehensive treatment of dyslipidemia is needed among FH patients to establish treatment goals. Raising awareness of the actual CVD risk, lifestyle improvement and improving compliance to statin therapy are promising strategies in reducing CVD risk among people with FH. There is a lack of evidence-based interventions that incorporate this comprehensive approach in the Netherlands as well as elsewhere. Our experiences with this intervention will be indicative about the suitability, feasibility and benefits of this approach for future interventions in other high-risk groups, such as Familial Combined Hypercholesterolemia (FCH) and diabetes.

The PRO-FIT project aims to develop such a comprehensive tailored lifestyle intervention and to evaluate this intervention in a randomized controlled trial, supported by a process and cost evaluation. In this article, we aim to outline the intervention and research design of the PRO-FIT project. PRO-FIT stands for promoting a healthy lifestyle in people with FH through an individually tailored lifestyle intervention.

## Methods/Design

### Development of the intervention

The PRO-FIT intervention was developed in a stepwise fashion, informed by a comprehensive theoretical framework and supported by an external advisory group. The advisory group brought together experts on behavioural change, computer tailored health education, and on FH and cardiovascular diseases. Their feedback and input was used to develop the intervention, and will be used during the intervention trial.

### Theoretical framework

The intervention of the PRO-FIT project was developed according to the integrated model for exploring motivational and behavioural change, the I-Change model (2.0)[20]. The core of the I-Change model is the Attitude-Social Influence-Self-efficacy (ASE) model which is comparable to the Theory of Planned Behaviour [21], but incorporates modelling and social support as social influences besides subjective norms. The I-Change model combines the ASE model with insight from stages of change models [24,25] and action planning models [26,27] to provide a comprehensive framework to study and facilitate behaviour change processes. It assumes that the behavioural change process can be distinguished in three phases: 1) Awareness, 2) Motivation and 3) Action. For each phase, specific change determinants have been proposed.

In the 'pre-motivational' awareness phase, people need to become aware of their risk behaviour. Important factors to proceed through this phase according to I-Change are knowledge, risk perceptions, and cues that prompt people to become aware. In the motivational phase, people need to become motivated to change their behaviour. Important factors in this phase according to the I-Change model are attitudes, social support and self-efficacy expectations. Proceeding through the motivational phase results in positive intention to change one's behaviour. In the action phase people need to translate intentions into actual behaviour change. In this phase several preparatory actions to facilitate behaviour change need to be planned and executed. People should convert their more global goal intentions into specific implementation intentions or action plans, with relevant strategies that will enable them to attain their goal.

**Figure 1:**
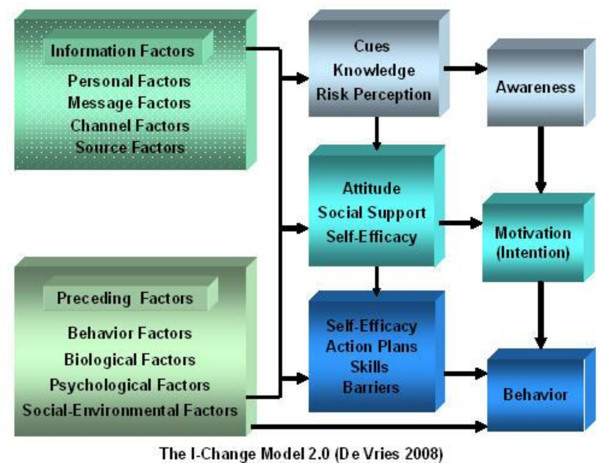
**I-Change model 2.0**. An integrated model for exploring motivational and behavioural change, used as theoretical framework during the development of the PRO-FIT intervention.

For a detailed overview of the I-Change model, see figure 1.

### Strategies

During the development of the PRO-FIT intervention, we aimed to focus on the earlier-mentioned factors of behavioural change identified in the I-Change model. A more detailed description of these factors, the strategies that will be employed, as well as the intervention components are outlined in table 1.

**Table 1:** Strategies to influence factors of behavioural change

Factors of behavioural change	Strategy
**Awareness factors **knowledge, risk perception and cues to action	Educating participants on their current CVD risk factors, with regard to their size and changeability (**risk communication**). Thereafter, translating this to behavioural change in their personal situation. Raising awareness by providing personal and normative behavioural feedback following **motivational interviewing **techniques.

**Predisposing factors **genetic predisposition, current lifestyle, personal characteristics and information factors.	**Tailoring **the communication of CVD risk factors and lifestyle counseling to the genetically predisposed risk of the participants and their personal characteristics (age, gender, members of the household) and their current lifestyle behaviour. A **multi-channel approach **is chosen, thereby offering the intervention by internet, face-to-face and by telephone.

**Motivational factors **attitude, social influence and self-efficacy	Giving personal feedback to participant's self-reported attitude and self-efficacy and by involving the social environment of the participant in making action plans.

**Ability factors**,	Stimulating participants to make action plans and discussing how to overcome possible barriers in behavioural change, thereby following **motivational interviewing **techniques.

### Risk communication

To raise awareness, participants will be presented with CVD risk information. Due to the predispositional character of the risk and its high dependency on medication use and current lifestyle behaviour, it is not possible to present participants with a valid, accurate personal numeral risk. Rather, participants will be presented with: 1)feedback on CVD risk behaviours to educate them about the contribution of these CVD risk factors to their overall CVD risk, 2)information on the changeability of these factors, and 3)cues about how these risk behaviours may be changed. The risk factors, their changeability and the cues to action will be presented to the participants on a personal webpage.

### Computer tailoring

Earlier research has shown that computer-tailored education is an innovative and promising method to motivate people to change their physical activity and dietary behaviours, and it has shown better effects than generic health education[23]. The fact that computer-tailored health education provides people with personalized feedback and advice is probably the main determinant of its effectiveness[24].

In this project, online tailored advice is focused on saturated fat intake, fruit and vegetables intake, physical activity, smoking behaviour and compliance to statin therapy. Online advice on saturated fat intake, physical activity and smoking behaviour is based on existing tailored information modules of the 'Healthy Life Check' (in Dutch: 'Gezondlevencheck') of the Netherlands Heart Foundation. This web-based computer-tailored lifestyle intervention was evaluated by Oenema et al [25] and reduced saturated fat intake and increased physical activity. Computer-tailoring is focused on personal performance level (current lifestyle behaviour), awareness of their own performance, as well as personal motivation to change, outcome expectations, attitude and self-efficacy. Since CVD risk reduction can be achieved by daily fruit and vegetable intake as well [19], online tailored advice modules on fruit and vegetables are added to the online tailored advice, mainly based on existing modules of the Live Healthy Coach (in Dutch: Leefgezondcoach) of the Dutch Diabetes Federation, developed at the Erasmus University Medical Center in Rotterdam, the Netherlands. Personalized feedback on compliance to statin therapy will be given through an existing tailored information module, tailoring on knowledge and personal beliefs about (the effect of) statin therapy, potential side effects of the prescribed drug and current compliance. This module was developed at the Rijksuniversiteit Groningen, the Netherlands. Additionally, a short-term plan and potential barriers to achieve this plan can be formulated in the online system.

These tailored advice modules are integrated into one online PRO-FIT*advice environment, a website that participants can visit using their personal account.

For the PRO-FIT computer-tailored intervention we have taken the main limitations into account identified in the main systematic review of effectiveness of computer-tailoring in the behavioural nutrition and physical activity field[23]. More specific, PRO-FIT combines web-based education with interpersonal counselling, and thereby combines repeated exposure to the intervention with individualization of messages and a social component.

### Motivational interviewing

Motivational interviewing (MI) was chosen as a technique to counsel the participants towards the desired behavioural change. MI was developed by Miller and Rollnick [26] and is a useful intervention strategy in behaviour change interventions[27]. MI is directive, but client-centred and its main goal is to facilitate the client in identifying and mobilizing his or her intrinsic values and goals related to the targeted behavioural changes. Meta-analyses indicate that MI can be effective in facilitating health behavioural changes across a range of domains[28,29].

The five main principles of MI are: 1)showing empathy, 2)avoiding discussion, 3)rolling with resistance, 4)supporting self-efficacy, and 5)raising awareness of a dissonance between actual behaviour and behavioural goals. The main interviewing strategies of MI are: asking open-ended questions, showing empathy, reflecting on the client, confirming and summarizing[26]. In this project, brief MI will be performed by a personal coach, a health professional trained in MI. MI will be done once face-to-face at the participants' home and five times by telephone. The main principles of MI will be used to develop interview protocols. For telephone counselling, an adjusted version of the telephone interview protocol of the Healthy Body Healthy Spirit trial will be used[30,31].

### The PRO-FIT intervention

The intervention consists of a personalized health counselling intervention. This is a combination of tailored web-based advice (PRO-FIT*advice) and face-to-face counselling complemented with telephone booster sessions (PRO-FIT*coach).

The goal of the intervention is to: 1) improve awareness of the cardiovascular disease risk through an increase of specific knowledge, cues to action and change in risk perception, 2) improve motivation with respect to healthy behaviour through an increase of specific knowledge and a change in attitude, self-efficacy and social influences, 3) adopt and maintain a healthier lifestyle, with regard to physical activity, saturated fat intake, fruit and vegetables intake, smoking and compliance to statin therapy, and 4) lower the level of LDL-C and other biological CVD risk indicators and thereby a reduction of the CVD risk.

#### Risk communication

Participants of the intervention group will receive a web link that directs them to the project website, where they can go through a number of web pages providing them with information about their CVD risk profile. After going through these web pages, they can log on to the tailored lifestyle advice (PRO-FIT*advice) with their personal username and password that are given in the email.

#### Computer tailoring

PRO-FIT*advice contains six advice modules on physical activity, fruit intake, vegetables intake, saturated fat intake, smoking and compliance to statin therapy. Participants can choose what modules to go through and in what order, but they will be advised and encouraged to complete all relevant modules (e.g. the module 'smoking' only if the participant is a smoker). For each module, participants first complete an online questionnaire that enables assessment of current behaviour and the relevant psychosocial correlates suggested by the I-Change model. After completion, the PRO-FIT*advice software will analyse the answers and create personalized feedback and behaviour change advice, provided on the computer screen. More specifically, feedback on current behaviour in accordance with national recommendations will be provided and, if the behaviour is not according to recommendations, suggestions will be given on how to make relevant behaviour changes. The participants will be encouraged to make a concrete action plan, i.e. to specify when, where and how they will make the changes as well as what preparatory actions are necessary.

#### Motivational interviewing

Two weeks after sending their personal PRO-FIT*advice username and password, participants will be visited at home by their lifestyle coach. The participant and the coach will further establish the level of the participant's knowledge about FH and cardiovascular risk factors, and risk perception in a personal counselling session assisted by the information from the risk communication web pages. Furthermore, the coach will have access to the participant's personal PRO-FIT*advice account and the advice will be discussed, ambivalence and barriers related to the recommended behaviour changes will be explored based on MI.

During the 12 months of follow-up one to five counsellor-initiated booster telephone sessions will be performed. The goal of these calls is twofold: to encourage the participant's current behavioural changes and to provide further brief motivational interviewing to encourage the planned behavioural changes. The number of telephone sessions will be based on the participant's action plans and their need for additional counselling. The calls will be scheduled with the participant and will be documented in the form of a personal calendar that is send to the participant with a small booklet in which all topics to be addressed during the counselling session will be listed.

Face-to-face counselling and telephone booster sessions will be performed by two trained lifestyle coaches. They received a special 3-day training programme on motivational interviewing techniques. For training purpose, during a pilot study, 20 pilot counselling sessions were scored by the coaches according to the Motivational Interviewing Skills Code (MISC)[32] and the Motivational Interviewing Treatment Integrity Code (MITI)[33]. According to these scores, and the experiences of the participants and the trainees, the quality of counselling was discussed and potential points of improvement were brought up. Each counselling session will be audio taped and both content and general characteristics will be documented into a database registration system. Both face-to-face and telephone counselling will be examined by two trained coders for fidelity to MI, using Motivational Interviewing Treatment Integrity (MITI 3.0) code[34]. To promote continued participation in the PRO-FIT trial, participants in both study groups will receive three incentives during the course of the trial.

### Pilot study

A pilot study to test the feasibility of the intervention and measurement content and procedures of the PRO-FIT trial was conducted in November 2008. Twenty participants from the target population were recruited for this pilot following the same recruitment strategies as are intended for the trial. All 20 participants completed the measurement and intervention in a one month time frame. Participants were interviewed and surveyed about their appreciation and logistics of and experiences with the measurement and intervention. Based on the pilot, minor adjustments were made in the content and procedure of both the measurement and intervention.

### Evaluation of the intervention

This PROFIT trial is designed as an RCT. Participants will be randomly assigned to either intervention or control group. In the intervention group, the participants will receive the comprehensive intervention as described above. Participants in the control group will receive usual care, which means that they will receive no intervention. Recruitment started in February 2009. Data collection continues until the summer of 2010.

The project design, procedures and informed consent form were approved by the Medical Ethics Committee of the VU University Medical Center (under registration number 2008/149), and all participants provide written informed consent.

### Study population

Participants are individuals who were diagnosed with FH by StOEH from January 1^st ^2007 to April 15^th ^2009. The invitation included an information brochure, a reply card, an informed consent letter and a reply envelope. Responders to the invitation are included in the project if they: 1) are aged 18-70 years, 2) are sufficiently fluent in Dutch, 3) have given informed consent, 4) have a LDL-C level that is >75^th ^percentile (corrected for age and gender), 5) live in a 150 km radius of Amsterdam, and 5) have access to the internet.

### Sample size

Information is lacking on the Standard Deviation (SD) of the mean intra-individual change in LDL-C, the main CVD risk indicator, over one year period of follow-up in a population that has recently been notified of their positive FH status. Being conservative, we expect the change to be large (35%). With an alpha of 0.05, the mentioned numbers (200 participants in intervention and 200 in control group) and an expected drop-out of 20%, the power is 90% to statistically detect an intervention effect of 9%. A 10% reduction of LDL-C is associated with a 13% reduced risk of major coronary events[35].

### Randomisation

A stratified computerized randomisation procedure will be carried out using Microsoft^© ^Office Access 2003 software. We will stratify participants according to cholesterol lowering medication use (yes/no), assuming that medication use implicates treatment by a general practitioner and/or medical specialist, who could have already given advice on lifestyle behaviour. In addition, we expect that a decrease in LDL-C because of the intervention is smaller if a participant already uses medication. After stratification, the randomisation procedure will be carried out.

If participants are members of the same household, cluster randomisation will be performed by clustering these participants and allocating them randomly to either the control or the intervention group. This will be done to prevent contamination of the intervention effect due to reciprocal communication about the intervention or control condition among participants. Stratification and randomization will be performed by an independent researcher, who is not involved in the intervention.

### Participant flow

**Figure 2:**
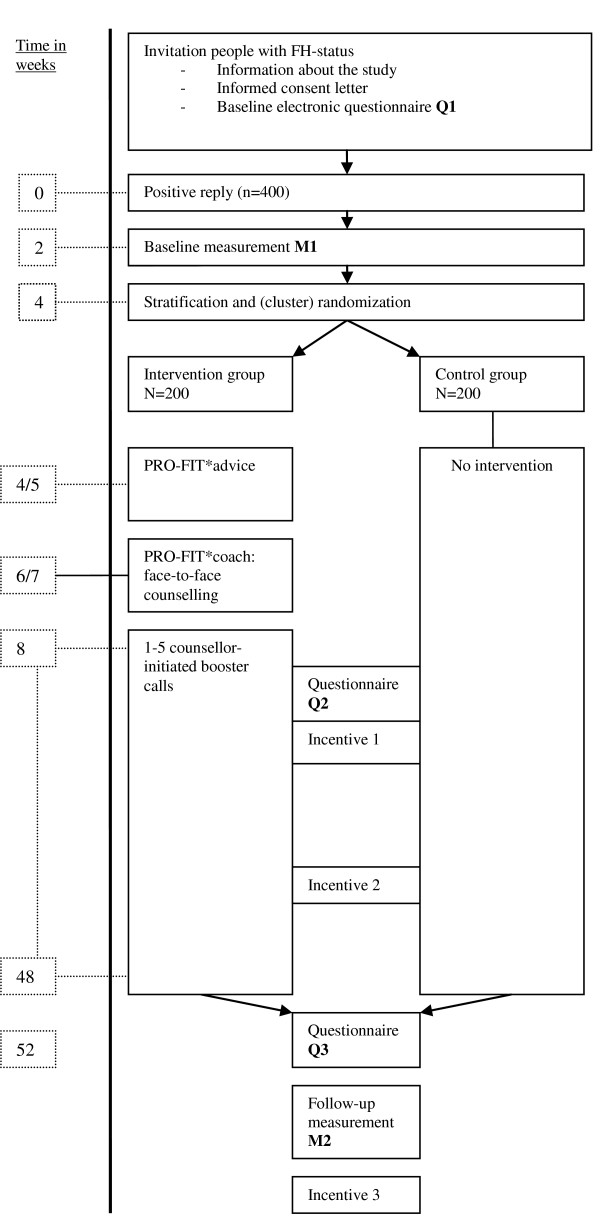
**Participant flow**. A detailed participant flow chart of the PRO-FIT project.

After the participant completed the reply card and signed the informed consent letter, baseline measurements will be performed and the baseline questionnaire will be sent out. Thereafter, 400 participants will be randomly assigned to either the intervention (n = 200) or control group (n = 200). Participants are followed for 12 months. For a detailed flow chart, see figure 2.

### Measurements

#### Biological CVD risk indicators

LDL-C, HDL-C and total cholesterol, triglycerides and glucose will be measured with fasting finger stick samples analyzed on a Cholestech LDX desktop analyzer (Cholestech, Hayward, USA). This portable analyzer is capable of providing a lipid profile (LDL-C-, HDL-C and TC, TC/HDL ratio and triglycerides) and glucose in approximately 5 minutes, using a lipid profile and glucose test cassette. The reproducibility and precision of lipids measurement by the LDX analyzer are within the guidelines of the NCEP[36,37]. The Cholestech LDX analyzer has been validated for point-of-care lipid measurements in clinical practice[38]. Blood pressure (in mmHg) is measured twice with a fully automated blood pressure monitor. The right arm is placed on a table and a cuff is placed on the right upper arm. Participants with a blood pressure of 140/90 mmHg or higher will be advised to visit their general practitioner. The mean value of the two measurements will be computed.

#### BMI and waist circumference

Height (in cm) will be measured on bare feet with a portable device with a wide measuring slide and a heel plate. Calibrated scales will be used to determine body weight (in kg) while participants wear light clothing only (e.g. underwear). Both weight and height will be measured twice, and the mean value of the two measurements will be computed and used to calculate BMI. Waist circumference (in cm) will be measured twice with a measurement tape to the nearest 0.1 cm, at the midpoint between the lower border of the ribs and the upper border of the pelvis. The mean value of the two measurements will be computed.

All measurements will be performed by a trained research assistant at the beginning of participation (M1 in figure 2) and after 12 months (M2 in figure 2).

### Questionnaires

#### Lifestyle behaviours

The level of physical activity will be measured by the Short QUestionnaire to ASsess Health-enhancing physical activity (SQUASH), which has been found to be fairly reliable (overall correlation: 0.58) and reasonably valid[39]. The focus regarding dietary intake is on saturated fat, fruit and vegetable intake, and will be measured by the short Dutch questionnaire on total and saturated fat intake[40,41] and on fruit and vegetable intake[42]. These questionnaires have been validated as related to seven day dietary records (47-49). For the fruit and vegetable questionnaire also biomarker validity was established[43]. Smoking behaviour will be assessed by a self-reported measure, asking participants if they are a current smoker, an ex-smoker, or a never smoker, how many years they smoke(d) and how many cigarettes or other tobacco products they smoke(d) a day[44].

#### Self-reported compliance to statin therapy

The five-item Medication Adherence Report Scale (MARS-5) will be used to measure self-reported compliance to statin therapy, which was found to have good reliability and validity[45]. In addition, pharmacy records will be used to study the persistence of medication use (period from first prescription to discontinuation) and refill compliance (percentage of prescribed medication that was actually obtained at the pharmacy). Permission to consult pharmacy records will be asked in the informed consent form.

#### Intention

Intention to change will be assessed with a self-report measure, asking participants: *Do you plan to change behaviour X*. The behaviour and the change was specified according to recommendations (e.g. raise the level of physical activity to >30 minutes a day) on a 5-point Likert scale ranging from *certainly yes *to *certainly no*. Additionally the participants will be asked how certain they were about acting upon their intention and *How sure are you of this? *(*absolutely sure *to *absolutely not sure*).

#### Risk perception

Leventhal's self-regulation model of illness cognition (SRM) provides a useful framework for considering and assessing the role of risk perception in response to communicating CVD risk to people with FH[46]. According to this model, a health threat activates a self-regulatory process where initially a coherent, common-sense understanding of the problem is developed. The cognitive illness-risk representations that are formed can be translated to the five core constructs of the SRM: identity, causal beliefs, timeline, consequences and control[47]. Questions on risk perception were developed from the literature and partly based on the brief Illness Perception Questionnaire (IPQ), the revised IPQ (IPQ-R)[48,49] and questionnaire of Claassen et al[50]. The constructs of the IPQ and IPQ-R are mainly based on the five elements of the SRM.

Participants will be asked if they actually know about their CVD risk and if they know how to reduce this risk, on a 5-point Likert scale ranging from *I totally disagree *to *I totally agree*. Emotional representation of CVD risk will be assessed questioning *When I think of my CVD risk, I feel... *on a 7-point Likert scale ranging from *not anxious at all *to *very anxious *and *not worried at all *to *very worried*.

The perceived CVD risk will be assessed questioning the comparative risk (*Of 100 men/women of my age, I think that approximately .... people will develop a CVD within the next 10 years*), the verbal perceived risk (*How likely is your chance of getting a CVD within the next 10 years?*) and verbal comparative risk (*According to you, what is your chance of developing a CVD within the next 10 years, compared to an average men/women of your age without FH?*) both on a 7-point Likert scale ranging from *very unlikely *to *very likely*. Additionally, we will assess perceived CVD risk from a personal point of view, questioning *For your own feeling, how big is your chance of developing a CVD within the next 10 years?' *on a 7-point Likert scale ranging from *very small *to *very big*. Finally, participants will be asked to score whether twenty possible causal beliefs (p.e. *I expect chance or bad luck as a potential cause for CVD*) for CVD were applicable to their situation. Furthermore, participants will rank what they thought were the three most important causes.

#### Psychosocial factors

Attitude, self-efficacy and social influence will be measured on a 5-point Likert scale ranging from (attitude) *very bad *to *very good*, (self-efficacy) *very difficult *to *very easy *and (social influence of partner, relatives, children, friends and/or experts) from *I totally agree *to *I totally do not agree*. Whether (a relative) having FH, (a relative) having CVD, an elevated CVD risk and/or death of a relative through CVD were cues to change lifestyle behaviour, will be measured on a 5-point Likert scale ranging from *I totally agree *to *I totally do not agree*. The psychosocial factors mentioned above will be measured with regard to all lifestyle behaviours, as well as to compliance to statin therapy. In addition, preference for autonomy will be measured with one item, asking *In general, when it comes to my health, I would rather have an expert tell me what I should do *on a 5-point Likert scale ranging from *I totally agree *to *I totally do not agree*. Response efficacy will be measured on a similar scale, assessing whether the participant believes statin treatment and lifestyle improvement are effective in reducing CVD risk.

Electronic questionnaires will be sent to the participants at the beginning of participation (Q1 in figure 2) and after 12 months (Q3). Additionally, risk perception will be measured after 3 months (Q2 in figure 2), and preference for autonomy will be measured after 12 months (Q3).

### Statistical analysis

Multiple linear and logistic regression analysis techniques will be performed. Potential confounders and effect modifiers (i.e. gender and age) will be checked. Data will be analysed according to the intention-to-treat principle.

### Process evaluation

A thorough systematic approach is chosen to monitor and document the implementation of the intervention during the trial. By using the RE-AIM framework, the translatability and public health impact of our project will be repeatedly evaluated by examining our work in light of the following five dimensions: 1) Reach among the target population; 2) Efficacy of the intervention; 3) Adoption by intermediaries; 4) Implementation - consistency of delivery of intervention; 5) Maintenance of intervention effects in individuals and populations over time[51]. Consequently, a structured process evaluation plan is developed according to Saunders[52]. A systematic approach is chosen to asses the implementation of the intervention, including recommended elements like fidelity, dose (delivered and received), reach, recruitment and context. Process evaluation questions are formulated on each element and accompany the questionnaire at 3 months (Q2) and at follow up (Q3).

### Economic evaluation

Economic evaluation consists of an analysis of differences in intervention development and implementation between the intervention and control group. The incremental costs of the intervention group compared to the control group will be divided by the incremental effect for the percentage improvement in LDL-C. The 95%-CI for these ratios is calculated using bootstrapping methods and they will be graphically presented on a cost-effectiveness plane. Utilities for the cost-utility analysis will be based on the EuroQol questionnaire[53], accompanying the questionnaire at baseline (Q1), at 3 months (Q2) and at follow-up (Q3).

## Discussion

In this article, we aim to outline the design of the PRO-FIT project, which is a RCT on the (cost-) effectiveness of a tailored lifestyle intervention to reduce the CVD risk of individuals with FH. The intervention is theory-driven and works systematically, aiming at a reduction of CVD risk through improvement of lifestyle behaviours and medication adherence.

Anticipated strengths of the approach include: it starts with aiming at accurate awareness of CVD risk, then giving evidence-based tailored feedback on lifestyle and finally, personally motivating people to move towards a healthy lifestyle. The social interaction during the face-to-face coaching session complements the single weakness of the individualized web-based approach, and thereby making this combination a promising one. Former research has shown that similar lifestyle interventions can effectively improve lifestyle behaviour[18].

Limitations of this project are: the study population is characterized by diversity and similarity at the same time. Both aspects can cause contamination of effects. Diversity is mainly due to the expected inter-participant differences at baseline, regarding biological CVD risk indicators, lifestyle behaviour, and use and type of medication. Similarity is caused by the familial nature of FH, resulting in a population with a high level of mutual communication. Both stratifying our sample on use of medication, and randomizing them in household clusters are methods to minimize potential contamination. However, these limitations should be considered during data analysis.

## Competing interests

The authors declare that they have no competing interests.

## Authors' contributions

KB, MvP, LK, JB and WvM provided support in the design of the study. KB coordinated the execution of the project and wrote the manuscript. All authors have read and corrected draft versions of the manuscript and approved the final manuscript.

## Pre-publication history

The pre-publication history for this paper can be accessed here:

http://www.biomedcentral.com/1471-2458/10/69/prepub
